# SDT: A Virus Classification Tool Based on Pairwise Sequence Alignment and Identity Calculation

**DOI:** 10.1371/journal.pone.0108277

**Published:** 2014-09-26

**Authors:** Brejnev Muhizi Muhire, Arvind Varsani, Darren Patrick Martin

**Affiliations:** 1 Department of Clinical Laboratory Sciences, University of Cape Town, Cape Town, South Africa; 2 School of Biological Sciences and Biomolecular Interaction Centre, University of Canterbury, Christchurch, New Zealand; 3 Department of Plant Pathology and Emerging Pathogens Institute, University of Florida, Gainesville, Florida, United States of America; Division of Clinical Research, United States of America

## Abstract

The perpetually increasing rate at which viral full-genome sequences are being determined is creating a pressing demand for computational tools that will aid the objective classification of these genome sequences. Taxonomic classification approaches that are based on pairwise genetic identity measures are potentially highly automatable and are progressively gaining favour with the International Committee on Taxonomy of Viruses (ICTV). There are, however, various issues with the calculation of such measures that could potentially undermine the accuracy and consistency with which they can be applied to virus classification. Firstly, pairwise sequence identities computed based on multiple sequence alignments rather than on multiple independent pairwise alignments can lead to the deflation of identity scores with increasing dataset sizes. Also, when gap-characters need to be introduced during sequence alignments to account for insertions and deletions, methodological variations in the way that these characters are introduced and handled during pairwise genetic identity calculations can cause high degrees of inconsistency in the way that different methods classify the same sets of sequences. Here we present Sequence Demarcation Tool (SDT), a free user-friendly computer program that aims to provide a robust and highly reproducible means of objectively using pairwise genetic identity calculations to classify any set of nucleotide or amino acid sequences. SDT can produce publication quality pairwise identity plots and colour-coded distance matrices to further aid the classification of sequences according to ICTV approved taxonomic demarcation criteria. Besides a graphical interface version of the program for Windows computers, command-line versions of the program are available for a variety of different operating systems (including a parallel version for cluster computing platforms).

## Introduction

The ever advancing rate at which novel viral genomes are being determined is creating a serious challenge both for taxonomists seeking to ensure the consistent and accurate classification of these genomes, and for laboratory virologists attempting to accurately name newly determined genome sequences prior to deposition into public sequence databases. Given that in many cases the only taxonomically useful information that is available for a particular genome sequence is the sequence data itself, the use of pairwise nucleotide sequence identity measures is becoming increasingly popular as a means of objectively classifying bacteria [1] and viruses [2], [3] into consistent and practically useful operational taxonomic units (OTUs) such as variants, strains, species or genera. In the case of many viruses which have small genomes (<30 kb long), whole genome sequences can be efficiently aligned, and genome-wide pairwise sequence identity scores are therefore used routinely for their functional classification. Accordingly, for over 50% of currently known virus families, the International Committee on Taxonomy of Viruses (ICTV) has, amongst other phylogenetic and biological criteria, endorsed the use of genome-wide nucleotide or amino acid sequence identity thresholds for the classification of novel virus isolates (according to ICTV proposals published since the 8^th^ ICTV Report; http://ictvonline.org/).

Despite the obvious appeal of using genetic identity scores between pairs of sequences to objectively classify these sequences, there is frequently a lack of clarity on exactly how such scores should be calculated. For example, given a new virus sequence and the desire to classify it based on an established ICTV approved species demarcation threshold, there are many different ways in which a researcher might determine whether or not it should be included within an already established species. Computer programs such as MUSCLE [4], CLUSTALW [5], MAFFT [6] or BLAST [7] could be used to make either multiple individual pairwise sequence alignments or a single multiple sequence alignment and other programs such as MEGA5 [8], PHYLIP [9], PAUP [10] or GENEIOUS (http://www.geneious.com/) could be used to calculate a variety of different pairwise identity scores. Unsurprisingly, for a given pair of sequences, different combinations of alignment and pairwise identity calculation approaches will in many cases yield a fairly broad range of possible sequence identity scores.

Whereas different alignment methodologies will very frequently infer different patterns of insertions and deletions (indels) during the evolutionary histories of any particular pair of sequences [11]–[13], independent pairwise alignments of sequences will tend to yield higher pairwise identity scores than those calculated for the same pairs of sequences within the context of multiple sequence alignments [13], [14]. Also, when calculating pairwise identity scores between any particular pair of sequences, the way in which indels are treated can have a very substantial impact on the identity scores that are calculated. Specifically, indel characters (usually “-”) that were inserted during multiple or pairwise sequence alignment might either be ignored or treated as a fifth character state. If indels are treated as a fifth character state then sites where both of the sequences being compared have indel characters might either be ignored or treated as matches (in which case they will inflate identity scores). Conversely, if sites where one but not the other sequence has an indel character are treated as mismatches the calculated identity scores will be lower than if such sites were ignored.

Particularly pertinent in the context of ever-increasing sequence database sizes is the fact that for any given pair of sequences, the differences between all these various alignment and identity score calculation approaches are expected to increase as the number of sequences that are being compared increases. This is because the computational complexity of accurately aligning multiple sequences increases exponentially with the number of sequences being aligned [15]. Put simply what this means is that as sequence numbers get larger multiple sequence alignments will tend to become more inaccurate. Although correction of alignments by eye is generally recommended for small datasets, it is not a practical option for datasets containing hundreds of sequences drawn from multiple different virus species. Alignment by eye is particularly undesirable in the context of taxonomic classification as it is both time-consuming and has the potential to seriously undermine the objectivity and consistency with which sequences are classified.

The pairwise identity calculation approaches that will be least impacted by these problems are those relying exclusively on independent pairwise alignments. Besides being unaffected by dataset sizes, pairwise alignment is computationally tractable: i.e. given a specified set of rules for penalising mismatches and inserting gap characters, the optimal pairwise alignment can always be found in a reasonable time [16]. Pairwise alignments also lack sites where both sequences have indel characters and are therefore far less affected by how indel characters are treated during identity score calculations. When calculating the identity scores of pairwise aligned sequences, the issue of gap character handling can be even further minimised by simply ignoring all sites at which a gap character is present in either one of the sequences being compared: an approach commonly adopted when calculating evolutionary distances in the context of phylogenetic tree construction [17], [18].

The demand for computational tools that will expedite the consistent and accurate classification of the increasing numbers of complete virus genomes deposited in public databases each year has prompted the development of computer programs such as PASC (PAirwise Sequence Comparison; [2]), and DEmARC (DivErsity pArtitioning by hieRarchical Clustering; [19]). Besides providing a means for virologists to accurately classify novel virus genome sequences at the species level prior to their publication, these tools have been especially useful both in the refinement of taxonomic classification criteria and for updating the classifications of hundreds of virus genome sequences that have been deposed in publically accessible sequence databases over the past three decades [2], [3], [20], [21].

PASC, the most widely used of these programs, is a web-based tool developed by the National Centre for Biotechnology Information (NCBI) [2]. Given a novel virus genome sequence, PASC compares this to a defined set of publicly available sequences and then uses either BLAST [7] similarity scores or Needleman-Wunsch (NW; [16]) pairwise-alignment based genetic identity scores to generate frequency distributions of pairwise genetic identity scores (based on both the input and database sequences). The output can then be used to either classify the input sequence or manually identify taxonomically optimal pairwise identity-based species or genus demarcation thresholds.

Rather than focusing on pairwise identity scores determined from multiple sequence alignments, DEmARC utilises multiple sequence alignments and model-based pairwise evolutionary distance calculations that ignore indel sites. In this regard, DEmARC is perhaps better suited to the objective identification of phylogenetically supported taxonomic demarcation criteria than for use by general virologists for the classification of new sequences based on pairwise identity-based classification criteria. It is also worth noting that while applicable to the analysis of nucleotide sequence data, DEmARC was specifically designed for the analysis of conserved amino acid sequence domains: an intended application that would substantially diminish alignment accuracy issues.

While both PASC and DEmARC are potentially very useful for the establishment of objective classification criteria and the refinement of existing virus classifications, in our opinion neither of the approaches is ideally suited for use by general virologists seeking to accurately and consistently classify the novel virus genomes that they sequence into either established ICTV approved species or strains or other functionally useful OTUs. Whereas DEmARC demands the analysis of carefully constructed and edited multiple sequence alignments, PASC forces users to scan a novel sequence against a representative selection of related sequences that is generally tailored specifically to classify genomes only down to the species level (i.e., the list of sequences in many cases excludes sequences that might be of interest for making strain, variant or other higher resolution OTU classifications). PASC also relies entirely on analysing sequences in the configuration in which they were submitted to the public sequence databases. This is particularly problematic because the NW pairwise alignment method implemented in PASC encounters difficulties when circular genome sequences have been deposited with inconsistent starting and ending coordinates. The developers of PASC have therefore recommended the use of a BLAST-based alignment comparison approach that is much less affected by this issue [2]. However, from a viral taxonomic classification perspective, there remains a potentially serious consistency issue when it comes to using BLAST scores instead of NW alignment-based pairwise identity scores. Specifically, in a given dataset containing both closely related and distantly related genome sequences, whereas BLAST similarity scores between the closely related sequences might be calculated across the entire genome length, the BLAST similarity scores for the more distantly related sequences may only be calculated across the portions of the sequences that are most conserved. Besides this consistency issue, there is also no obvious way to translate BLAST scores into genome-wide pairwise identity scores: i.e. the intuitively obvious measure of genome-wide similarity that is generally used by the ICTV in their classification guidelines and is generally overwhelmingly preferred by general virologists when describing the genetic relatedness of virus isolates.

Here we present Sequence Demarcation Tool for Windows (SDT version 1.2, www.cbio.uct.ac.za/SDT), a stand-alone program with a simple user friendly graphical interface. Rather than being targeted at hard-core virus taxonomists, SDT is specifically targeted at laboratory and field virologists wanting to rapidly and consistently use the pairwise identity-based ICTV taxonomic guidelines to tentatively classify new viral genome sequences. Although the program has been recently used for the reclassification of viruses in the family *Geminiviridae* [3], [20], [22]–[31], in the classification of viruses in the families *Circoviridae* [32] and *Nanoviridae* [33], the characterisation of novel highly divergent viral genomes sampled during metagenomic surveys [34], [35], and the comparison of protein sequence similarities in already characterised viruses species [36]–[39] and novel viruses [34], [35], [37]–[45]. SDT is functionally similar to PASC in that it objectively applies a robust NW-based pairwise alignment approach with a pairwise identity calculation that ignores alignment positions containing indel characters. The primary differences between SDT and PASC are that: (1) it is not restricted to using predefined sets of sequences, (2) it is geared specifically to the objective taxonomic classification of new virus sequences within the context of ICTV endorsed pairwise identity based strain, species and genus demarcation thresholds, and (3) it can produce publication quality colour coded pairwise-identity matrices with sequences ordered according to their degrees of phylogenetic relatedness. We also provide both command-line versions of SDT for Linux (SDT_Linux) and MacOS (SDT_MacOS), and a parallel Message Passing Interface based version for Linux (SDTMPI_Linux) that can be used on high performance computing clusters.

## Materials and Methods

### Implementation of SDT

A graphical user interface for SDT (available at www.cbio.uct.ac.za/SDT), is implemented in Visual Basic 6.0 and runs on Windows XP, 7 and 8. Command-line versions of SDT, SDT_Linux and SDT_MacOS and a parallel version, SDTMPI_Linux are provided for both 32 and 64 bit operating systems and are all written in python. While SDT has a graphical user interface that is complete with data visualisation tools, the command-line versions only produce numerical data. However, all these versions apply the same sequence identity calculation procedures.

### Sequence identity calculation

**Figure 1:**
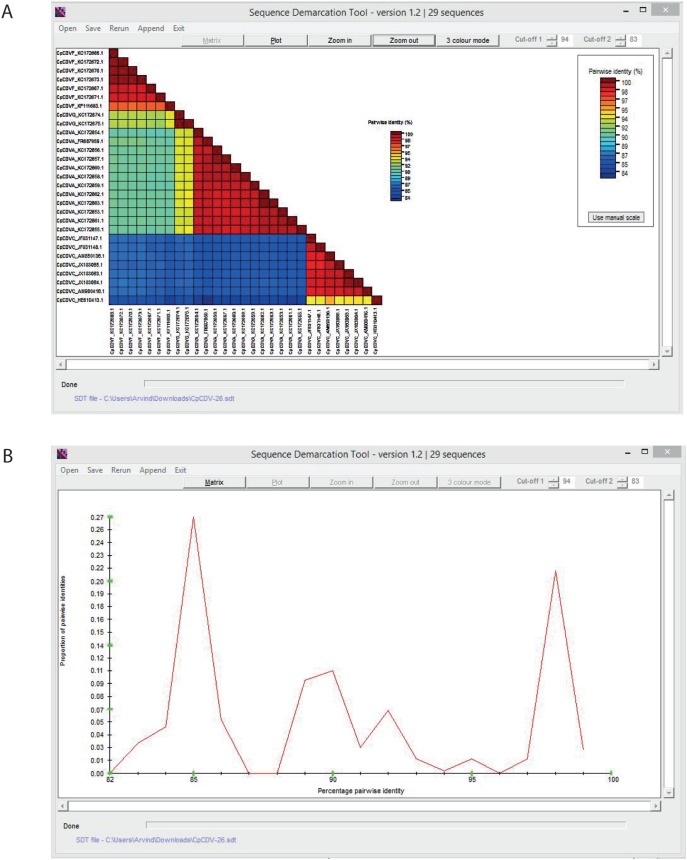
The SDT interface. (**A**) Colour-coded pairwise identity matrix generated from 29 *Chickpea chlorotic dwarf virus* genomes. Each coloured cell represents a percentage identity score between two sequences (one indicated horizontally to the left and the other vertically at the bottom). A coloured key indicates the correspondence between pairwise identities and the colours displayed in the matrix. (**B**) Pairwise identity frequency distribution plot. The horizontal axis indicates percentage pairwise identities, and the vertical axis indicates proportions of these identities within the distribution. While peaks on the graph indicate pairwise sequence identity thresholds that would yield the most ambiguous classifications, troughs indicate thresholds that would yield the least ambiguous classifications and could therefore be tentatively used as relatively conflict free operational taxonomic unit demarcation cut-offs.

Given an input FASTA file, SDT aligns every unique pair of sequences (S sequences yield [S×(S-1)]/2 alignments) using the NW algorithms implemented in MUSCLE [4], ClustalW [5] or MAFFT [6] (the user can choose whichever program he/she prefers), and computes the identity score for each pair of sequences as 1-M/N, where M is the number of mismatched nucleotides and N is the total number of columns along the alignment where neither sequence has a gap character. The program then uses the NEIGHBOR component of PHYLIP [9] to generate a rooted neighbour-joining phylogenetic tree of sequences according to which computed scores are rearranged so as to order sequences according to their likely degrees of evolutionary relatedness. Finally, SDT generates a frequency distribution of pairwise-identities. The graphical program interface (Figure 1) provides both publication quality visualisations of results and enables results to be saved in a variety of graphical and numerical data file formats.

### Pairwise identity matrix and pairwise identity distribution plots

SDT displays pairwise identity scores using a colour-coded matrix (Figure 1A) which provides more intuitively accessible insights into the overall relationships between sequences in a dataset than the tables of pairwise sequence identity scores that are widely used for this purpose. The colours in this matrix can be adjusted to reflect, for example, an ICTV species demarcation criterion such that identities between sequences that are over the threshold are represented in a shade of one colour whereas those that fall below the threshold are represented in a shade of a different colour. The ordering of sequences along the axes of the matrix reflects the ordering of the sequences as they would appear in a neighbour joining phylogenetic tree: i.e. the pairwise identities between sequences are clustered within the matrix in an evolutionarily meaningful way. This makes it very easy to check exactly which groups of sequences a novel sequence is most closely related to and, depending on the colours of the cells in the matrix, immediately indicates which genus, species, strain or other operational taxonomic unit it could most appropriately be assigned to. For a detailed example of how SDT pairwise identity matrices can be applied to the classification of novel virus genomes please refer to [3] and [22].

SDT also produces plots of the pairwise identity score frequency distribution which, like similar plots produced by PASC and DEmARC, are useful in guiding the establishment of taxonomic demarcation criteria (Figure 1B). Whereas peaks in such plots indicate pairwise identity thresholds that would yield a maximum number of ambiguous classifications (something which is undesirable), troughs in these plots indicate pairwise identity thresholds that would yield a minimum number of ambiguous classifications (something which is desirable). The colour coded matrix and pairwise identity distribution plots can both be saved either as bitmap images or as scalable and editable high-resolution graphic files in enhanced metafile format that are suitable for publication. For a detailed example of how SDT pairwise identity distribution plots can be applied to the establishment of novel virus genomes please refer to [3] and [22].

### Usage of pre-computed identity scores

When the computations are finalised, all versions of SDT allow a completed analysis session to be saved to a SDT readable file (with file extension “.sdt”) which subsequently can be reloaded. Upon reloading such a file in SDT, the program allows the addition of new sequences and then only computes scores for those sequence pairs that include the newly added sequences. Doing this vastly speeds up the analysis of new sequences and allows a user to very efficiently grow the size of project specific datasets.

### Creation of Datasets based on sequence identities

Given a set of input sequences and their corresponding pairwise sequence identity scores it is possible for SDT to objectively generate datasets comprising sequences of a desired level of diversity/identity that are tailored to further genome evolution analyses such as inference of patterns of natural selection or the identification of conserved genomic secondary structures [32], [46]. Once sequence identity scores are computed, SDT provides an efficient way to generate such datasets. All that is required of the user is to indicate a minimum and a maximum identity percentage and the program will then partition the input sequence dataset into sets of non-overlapping sequence files, with each file containing only sequence pairs with identities that are within the user specified range.

### The SDT_Linux, SDT_MacOS and SDTMPI_Linux command line versions

The python coded command-line versions of SDT for Linux, MacOS and high performance computing clusters are ideal for inclusion within automated sequence classification pipelines. These versions apply precisely the same sequence identity calculation approach as SDT but only generate pairwise identity scores in various comma separated value (CSV) text formats. Although there is no graphical output from these versions, the CSV files that are generated are formatted such that a colour coded pairwise identity matrix and distribution plot can easily be constructed using the R programming language (www.r-project.org) or MATLAB (http://www.mathworks.com/products/matlab/). Also, the.sdt formatted files that are generated by these versions of the program can be opened in the graphical interface version of the program to produce colour-coded distance matrices and pairwise identity plots. Whereas the SDT_Linux, SDT_MacOS and SDTMPI_Linux versions all require that python (available from https://www.python.org) be installed on the machines on which they are run, the SDTMPI_Linux version additionally requires the installation of the Python Message Passing Interface library (MPI4PY; available at http://mpi4py.scipy.org/docs/usrman/install.html).

### Comparison of SDT performance with alternative sequence comparison methods

For an objective comparison of SDT’s consistency with that of alternative pairwise sequence comparison methods, we used SDT and DEmARC to analyse the same set of 25 mastrevirus full genome sequences within the context of progressively increasing dataset sizes. Although it was not possible to run this test with PASC (due to the stringent sequence input requirements of this program), it is anticipated that PASC would have exactly the same degree of consistency as SDT (it too relies on pairwise sequence alignments). A dataset of 400 mastrevirus full genome sequences (Dataset S1), was progressively subdivided to generate five sub-alignments of 200, 100, 50 and 25 sequences, all containing the same set of 25 sequences. These were all analysed unaligned by SDT which produced pairwise identity scores for each of the 300 pairwise comparisons between the 25 sequences common to all five datasets. The identity scores once produced were converted to Hamming distances by subtracting them from one (so as to enable a more direct comparison with DEmARC). After aligning each individual dataset using MUSCLE (with default settings), Hamming genetic distances and DEmARC evolutionary distances were calculated for each of the same 300 pairwise sequence comparisons in each of the five alignments.

### Comparison of parallel and serial versions of SDT

We analysed 1000 publically available begomovirus sequences (Dataset S2; requiring 499,500 pairwise sequence alignments of ∼2800nts; Table 1) with 32 and 64 bit versions of SDTMPI_Linux and SDT_Linux using MUSCLE to perform pairwise alignments. The 32 and 64 bit versions of SDT_Linux were run on a 2.8 GHz computer with 24 GB of RAM (with this 32 bit version by definition being restricted to using <2 GB of RAM), and the 32 bit and 64 bit versions of SDTMPI_Linux were run on 20 or 40 cores each running at 2.8 GHz with 24 GB of RAM (again with the 32 bit version being restricted to using <2 GB of RAM).

## Results and Discussion

### The consistency of SDT relative to alternative virus classification tools

**Figure 2:**
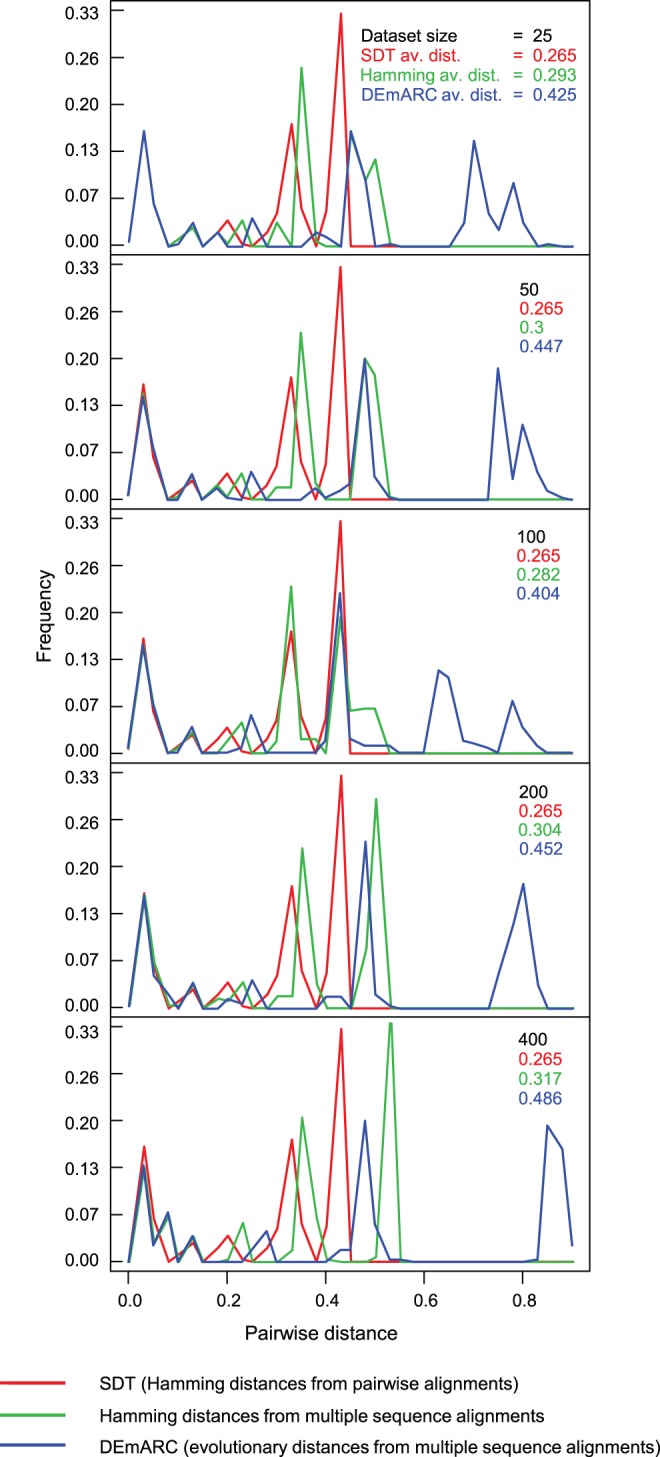
Distribution of pairwise genetic/evolutionary distances of the same set of 25 mastrevirus full genome sequences in the context of progressively larger sequence datasets. The constant frequency distribution (represented by red graph) illustrates the consistency of pairwise distance calculation based on pairwise alignments while the changing frequency distributions (represented by blue and green graphs) indicate how pairwise distance scores based on multiple sequence alignment tend to become inflated as dataset sizes get larger.

Although all of the pairwise comparison methods produced very similar results for sequences sharing between 90 and 100% pairwise identity, distinct differences between the methods were clearly observable in all datasets for sequence pairs sharing less than 80% identity (Figure 2). This observation is expected since sequence alignment only becomes non-trivial (and hence more error prone) when some of the sequences being aligned have accumulated multiple nucleotide substitution, insertion and deletion mutations since their most recent common ancestors.

For all datasets SDT yielded identical pairwise identity score distributions whereas the distributions yielded by the multiple sequence alignment-based methods differed substantially between the different datasets. This indicates that SDT is absolutely consistent whereas the other methods are not. It should be pointed out here that the absolute consistency of SDT is an obvious consequence of it using pairwise sequence alignments rather than multiple sequence alignments. In this regard it is absolutely certain that PASC too would have been found to be similarly consistent had it been flexible enough to allow the analysis of the various datasets.

Other points that should be noted in Figure 2 are that, firstly, the multiple alignment-based comparison methods always yielded higher average distance estimates than SDT, and secondly, that the magnitudes of these differences tend to increase with increasing dataset size (with the 100 sequence alignment being a notable exception). These observations simply confirm that pairs of sequences in the context of multiple sequence alignments tend to appear less similar to one another than they do in the context of pairwise sequence alignments.

It is important to point out here that the higher degrees of identity inferred by SDT do not necessarily imply that SDT identity estimates are more accurate than those inferred from the multiple sequence alignments. It is entirely plausible that, relative to the gap characters inserted during the pairwise alignment of two sequences, the positions of gap characters within pairs of sequences that are drawn from a multiple sequence alignment might better reflect the patterns of insertion and deletion that actually occurred during the evolution of the sequences. It is in fact expected that identity estimates based on pairwise alignments could at least slightly overestimate the relatedness of sequences: for example, even two completely random sequences can yield pairwise identity scores of >40% following pairwise alignment. In the context of virus classification, however, this is not necessarily a bad quality: particularly in a publication environment that incentivises the discovery of novel virus genera, species and strains. If anything, slightly overestimating pairwise identity estimates will force a degree of conservatism when proposing that new taxonomic groupings be created to accommodate novel virus isolates.

### Speed gains of SDT with parallelisation

In addition to the graphical version of SDT being extremely easy for non-specialists to use (it is very difficult to even purposefully manipulate the program to yield inflated or deflated identity scores), the software is also flexible enough to be of interest to more specialist users. For example, the command line versions can be directly slotted into analysis pipelines to automatically identify rational operational taxonomic unit demarcation thresholds and then automatically apply these to the subdivision of large datasets for downstream analyses. In this regard the SDTMPI_Linux version of SDT was specifically designed for the analysis of large datasets (containing more than 1000 sequences) and is well suited for inclusion in high throughput viral metagenome sequencing pipelines. The improvement in analysis speed afforded by SDTMPI_Linux over SDT_Linux was illustrated by an analysis of 1000 begomovirus sequences (requiring 499,500 pairwise sequence alignments of ∼2800 nts; Table 1). The 32 bit version of SDT_Linux took 3740.37 min (∼62.34 h) whereas SDTMPI_Linux running on 20 cores (each with similar specifications to that used with the serial version) took 188.56 min (∼3.14 h). SDTMPI_Linux running on 40 cores took only 96.63 min (1.61 h). The speed-up improvements with 20 and 40 cores were therefore 19.8 and 38.7 fold, respectively. Overall the 64 bit version of SDT yielded a further 13% increase in speed which is likely due to more efficient memory use. The 64 bit version of SDT would likely yield even better performance gains over the 32 bit version when analysing longer sequences (Table 1).

**Table 1:** Speed-ups achieved with the parallelised versions of SDT.

System	Program	Numberof Sequences	Numberof cores	Processor speed(GHz)	RAM(GB)	Time(min.)	Time (hrs.)	Speed up
32 bit	SDT_Linux	1000	1	2.8	24	3740.37	62.34	
	SDTMPI_Linux	1000	20	2.8	24	188.56	3.14	19.8 fold
	SDTMPI_Linux	1000	40	2.8	24	96.63	1.61	38.7 fold
64 bit	SDT_Linux	1000	1	2.8	24	3343.37	55.72	
	SDTMPI_Linux	1000	20	2.8	24	173.02	2.76	19.3 fold
	SDTMPI_Linux	1000	40	2.8	24	85.43	1.42	39.1 fold

## Conclusions

We present a free open-source cross-platform computer program that has been specifically designed to enable general virologists to consistently classify newly determined virus full genome sequences according to ICTV endorsed pairwise genetic identity based genus, species and strain demarcation recommendations. Besides providing the means to minimise inconsistencies in virus taxonomic classifications, the program is suitable for use both by biologists with limited computational skills and more computationally capable biologists that require the rapid automated analysis of very large datasets. Unlike the similar sequence classification tool, PASC, SDT is not exclusively designed for full virus genome based pairwise identity calculations but is also usable as an amino acid sequence classifier – a fact which could make it very useful for the characterisation of novel highly divergent viruses.

Although we have primarily focused here on the merits of SDT relative to PASC and DEmARC, it should be stressed that SDT is not a competitor of PASC and DEmARC – it is instead a complementary tool that could be used in conjunction with these other methods to establish and effectively implement pairwise identity based virus classification systems. For example DEmARC might be used by the ICTV to establish a solid evolutionary rationale for defining a particular set of species, PASC might then be used by individual ICTV working groups to establish easy to apply pairwise identity thresholds that optimally conform with the DEmARC classifications, and SDT (or equivalent software) might be used by individual virologists to consistently apply these thresholds during the tentative classification of novel virus isolates that they submit to public sequence databases. Finally, even if SDT is not deemed suitable as a classification tool by particular ICTV working groups, it will still have widespread utility as a tool for graphically visualising colour coded pairwise genetic similarities of large numbers of sequences – a niche that is currently unfilled by any other sequence analysis software.

The various versions of SDT that have been described here, along with instructions for their installation and use, are freely available at www.cbio.uct.ac.za/SDT.

## Supporting Information

Dataset S1 **Full genome sequences used to compare SDT to other methods.**
(FAS)

Dataset S2 **Full genome sequences used to assess the speed gained with parallelisation of SDT.**
(FAS)

## References

[1] Kim M, Oh H-S, Park S-C, Chun J (2014) Towards a taxonomic coherence between average nucleotide identity and 16 S rRNA gene sequence similarity for species demarcation of prokaryotes. Int J Syst Evol Microbiol 64: 346–351. 24505072 10.1099/ijs.0.059774-0

[2] Bao Y, Chetvernin V, Tatusova T (2012) PAirwise Sequence Comparison (PASC) and its application in the classification of filoviruses. Viruses 4: 1318–1327. 23012628 10.3390/v4081318 PMC3446765

[3] Muhire B, Martin DP, Brown JK, Navas-Castillo J, Moriones E, et al (2013) A genome-wide pairwise-identity-based proposal for the classification of viruses in the genus Mastrevirus (family Geminiviridae). Arch Virol 158: 1411–1424. 23340592 10.1007/s00705-012-1601-7

[4] Edgar RC (2004) MUSCLE: multiple sequence alignment with high accuracy and high throughput. Nucleic Acids Res 32: 1792–1797. 15034147 10.1093/nar/gkh340 PMC390337

[5] Larkin MA, Blackshields G, Brown NP, Chenna R, McGettigan PA, et al (2007) Clustal W and Clustal X version 2.0. Bioinformatics 23: 2947–2948. 17846036 10.1093/bioinformatics/btm404

[6] Katoh K, Standley DM (2013) MAFFT multiple sequence alignment software version 7: improvements in performance and usability. Mol Biol Evol 30: 772–780. 23329690 10.1093/molbev/mst010 PMC3603318

[7] Altschul SF, Gish W, Miller W, Myers EW, Lipman DJ (1990) Basic local alignment search tool. J Mol Biol 215: 403–410. 2231712 10.1016/S0022-2836(05)80360-2

[8] Tamura K, Peterson D, Peterson N, Stecher G, Nei M, et al (2011) MEGA5: molecular evolutionary genetics analysis using maximum likelihood, evolutionary distance, and maximum parsimony methods. Mol Biol Evol 28: 2731–2739. 21546353 10.1093/molbev/msr121 PMC3203626

[9] PHYLIP (Phylogeny Inference Package) Version 3.57c (1995)

[10] Swofford DL (2002) PAUP*: Phylogenetic Analysis Using Parsimony (*and Other Methods). Version 4. Sunderland, Massachusetts: Sinauer Associates, Inc.

[11] Thompson JD, Plewniak F, Poch O (1999) A comprehensive comparison of multiple sequence alignment programs. Nucleic Acids Res 27: 2682–2690. 10373585 10.1093/nar/27.13.2682 PMC148477

[12] Wilm A, Mainz I, Steger G (2006) An enhanced RNA alignment benchmark for sequence alignment programs. Algorithms Mol Biol 1: 19 doi:10.1186/1748-7188-1-19 17062125 10.1186/1748-7188-1-19 PMC1635699

[13] Katoh K, Kuma K, Toh H, Miyata T (2005) MAFFT version 5: improvement in accuracy of multiple sequence alignment. Nucleic Acids Res 33: 511–518. 15661851 10.1093/nar/gki198 PMC548345

[14] Sievers F, Dineen D, Wilm A, Higgins DG (2013) Making automated multiple alignments of very large numbers of protein sequences. Bioinformatics 29: 989–995. 23428640 10.1093/bioinformatics/btt093

[15] Elias I (2006) Settling the intractability of multiple alignment. J Comput Biol 13: 1323–1339. 17037961 10.1089/cmb.2006.13.1323

[16] Needleman SB, Wunsch CD (1970) A general method applicable to the search for similarities in the amino acid sequence of two proteins. J Mol Biol 48: 443–453. 5420325 10.1016/0022-2836(70)90057-4

[17] Felsenstein J (2004) Inferring Phylogenies. Sunderland, Massachusetts: Sinauer Associates, Inc.

[18] Lemey P, Salemi M, Vandamme A, editors (2009) The Phylogenetics Handbook, A Practical Approach to Phylogenetic Analysis and Hypothesis Testing. Second Edi. New York: Cambridge University Press.

[19] Lauber C, Gorbalenya AE (2012) Partitioning the genetic diversity of a virus family: approach and evaluation through a case study of picornaviruses. J Virol 86: 3890–3904. 22278230 10.1128/JVI.07173-11 PMC3302503

[20] Fiallo-Olivé E, Chirinos DT, Geraud-Pouey F, Moriones E, Navas-Castillo J (2014) Complete genome sequence of Jacquemontia yellow mosaic virus, a novel begomovirus from Venezuela related to other New World bipartite begomoviruses infecting Convolvulaceae. Arch Virol. 159: 1857–1860. 10.1007/s00705-014-1996-4 24463954

[21] Matthijnssens J, Ciarlet M, Heiman E, Arijs I, Delbeke T, et al (2008) Full genome-based classification of rotaviruses reveals a common origin between human Wa-Like and porcine rotavirus strains and human DS-1-like and bovine rotavirus strains. J Virol 82: 3204–3219. 18216098 10.1128/JVI.02257-07 PMC2268446

[22] Varsani A, Martin DP, Navas-Castillo J, Moriones E, Hernández-Zepeda C, et al (2014) Revisiting the classification of curtoviruses based on genome-wide pairwise identity. Arch Virol (158) 1873–82. 10.1007/s00705-014-1982-x 24463952

[23] Manzoor MT, Ilyas M, Shafiq M, Haider MS, Shahid AA, et al (2014) A distinct strain of chickpea chlorotic dwarf virus (genus Mastrevirus, family Geminiviridae) identified in cotton plants affected by leaf curl disease. Arch Virol. 159: 1217–1221. 10.1007/s00705-013-1911-4 24212888

[24] Kanakala S, Verma HN, Vijay P, Saxena DR, Malathi VG (2013) Response of chickpea genotypes to Agrobacterium-mediated delivery of Chickpea chlorotic dwarf virus (CpCDV) genome and identification of resistance source. Appl Microbiol Biotechnol 97: 9491–9501. 23955474 10.1007/s00253-013-5162-9

[25] Paz-Carrasco LC, Castillo-Urquiza GP, Lima ATM, Xavier CAD, Vivas-Vivas LM, et al.. (2014) Begomovirus diversity in tomato crops and weeds in Ecuador and the detection of a recombinant isolate of rhynchosia golden mosaic Yucatan virus infecting tomato. Arch Virol. doi:10.1007/s00705-014-2046-y. 10.1007/s00705-014-2046-y 24623091

[26] Du Z, Chen M, Wang Z, Liu Y, Zhang S, et al (2014) Isolation and molecular characterization of a distinct begomovirus and its associated betasatellite infecting Hedyotis uncinella (Hook. et Arn.) in Vietnam. Virus Genes 80: 246–250. 10.1007/s11262-014-1043-2 24510306

[27] Varsani A, Navas-Castillo J, Moriones E, Hernández-Zepeda C, Idris A, et al.. (2014) Establishment of three new genera in the family Geminiviridae: Becurtovirus, Eragrovirus and Turncurtovirus. Arch Virol. doi:10.1007/s00705-014-2050-2. 10.1007/s00705-014-2050-2 24658781

[28] Gharouni Kardani S, Heydarnejad J, Zakiaghl M, Mehrvar M, Kraberger S, et al (2013) Diversity of beet curly top Iran virus isolated from different hosts in Iran. Virus Genes 46: 571–575. 23329008 10.1007/s11262-013-0875-5

[29] Razavinejad S, Heydarnejad J, Kamali M, Massumi H, Kraberger S, et al (2013) Genetic diversity and host range studies of turnip curly top virus. Virus Genes 46: 345–353. 23225113 10.1007/s11262-012-0858-y

[30] Oluwafemi S, Kraberger S, Shepherd D, Martin D, Varsani A (2014) A high degree of African streak virus diversity within Nigerian maize fields includes a new mastrevirus species from Axonopus compressus. Arch Virol. doi:10.1007/s00705-014-2090-7. 10.1007/s00705-014-2090-7 24796552

[31] Srivastava A, S. Kumar S, Raj SK, Pande SS (2014) Association of a distinct strain of hollyhock yellow vein mosaic virus and Ludwigia leaf distortion betasatellite with yellow vein mosaic disease of hollyhock (Alcea rosea) in India. Arch Virol. DOI 10.1007/s00705-014-2108-1. 10.1007/s00705-014-2108-1 24810100

[32] Stenzel T, Piasecki T, Chrząstek K, Julian L, Muhire BM, et al (2014) Pigeon circoviruses display patterns of recombination, genomic secondary structure and selection similar to those of Beak and feather disease viruses. J Gen Virol. 95: 1338–1351. 10.1099/vir.0.063917-0 24639400

[33] Grigoras I, Del Cueto Ginzo AI, Martin DP, Varsani A, Romero J, et al (2014) Genome Diversity and Evidence of Recombination and Reassortment in Nanoviruses from Europe. J Gen Virol (95) 1178–1191. 10.1099/vir.0.063115-0 24515973

[34] Sikorski A, Massaro M, Kraberger S, Young LM, Smalley D, et al (2013) Novel myco-like DNA viruses discovered in the faecal matter of various animals. Virus Res 177: 209–216. 23994297 10.1016/j.virusres.2013.08.008

[35] Dayaram A, Potter KA, Moline AB, Rosenstein DD, Marinov M, et al (2013) High global diversity of cycloviruses amongst dragonflies. J Gen Virol 94: 1827–1840. 23596268 10.1099/vir.0.052654-0

[36] Phelps NBD, Mor SK, Armien AG, Batts W, Goodwin AE, et al (2014) Isolation and Molecular Characterization of a Novel Picornavirus from Baitfish in the USA. PLoS One 9: e87593. 24586283 10.1371/journal.pone.0087593 PMC3931614

[37] Liu Q, Zhang Z, Li Z, Qiao Qi, Qin Y, et al. (2014) Complete genome sequence of a novel monopartite begomovirus infecting sweet potato in China. Arch Virol. DOI 10.1007/s00705-013-1958-2. 10.1007/s00705-013-1958-2 24378821

[38] Tabasinejad F, Jafarpour B, Zakiaghl M, Siampour M, Rouhani H, et al.. (2014) Genetic structure and molecular variability of potato virus M populations. Arch Virol. DOI 10.1007/s00705-014-2037-z. 10.1007/s00705-014-2037-z 24658780

[39] Liu P, Li Z, Song S, Wu Y, (2014) Molecular variability of Apple chlorotic leaf spot virus in Shaanxi, China. Arch Virol. DOI 10.1007/s12600-013-0381-2.

[40] Bernardo P, Golden M, Akram M, Naimuddin, Nadarajan N, et al (2013) Identification and characterisation of a highly divergent geminivirus: evolutionary and taxonomic implications. Virus Res 177: 35–45. 23886668 10.1016/j.virusres.2013.07.006

[41] Piasecki T, Harkins GW, Chrząstek K, Julian L, Martin DP, et al (2013) Avihepadnavirus diversity in parrots is comparable to that found amongst all other avian species. Virology 438: 98–105. 23411008 10.1016/j.virol.2013.01.009

[42] Du Z, Tang Y, Zhang S, She X, Lan G, et al (2013) Identification and molecular characterization of a single-stranded circular DNA virus with similarities to Sclerotinia sclerotiorum hypovirulence-associated DNA virus 1. Arch Virol. 159: 1527–1531. 10.1007/s00705-013-1890-5 24318574

[43] Dayaram A, Galatowitsch M, Harding JS, Argüello-Astorga GR, Varsani A (2014) Novel circular DNA viruses identified in Procordulia grayi and Xanthocnemis zealandica larvae using metagenomic approaches. Infect Genet Evol 22: 134–141. 24462907 10.1016/j.meegid.2014.01.013

[44] Varsani A, Kraberger S, Jennings S, Porzig EL, Julian L, et al (2014) A novel papillomavirus in Adelie penguin (Pygoscelis adeliae) faeces sampled at the Cape Crozier colony, Antarctica. J Gen Virol. 95: 1352–1365. 10.1099/vir.0.064436-0 24686913

[45] Zawar-Reza P, Argüello-Astorga GR, Kraberger S, Julian L, Stainton D, et al (2014) Diverse small circular single-stranded DNA viruses identified in a freshwater pond on the McMurdo Ice Shelf (Antarctica). Infect Genet Evol 26: 132–138. 24859088 10.1016/j.meegid.2014.05.018

[46] Muhire BM, Golden M, Murrell B, Lefeuvre P, Lett J, et al (2014) Evidence of pervasive biologically functional secondary structures within the genomes of eukaryotic single-stranded DNA viruses. J Virol 88: 1972–1989. 24284329 10.1128/JVI.03031-13 PMC3911531

